# Use of Delphi in health sciences research: A narrative review

**DOI:** 10.1097/MD.0000000000032829

**Published:** 2023-02-17

**Authors:** Zhida Shang

**Affiliations:** a Faculty of Medicine and Health Sciences, McGill University, Montreal, QC, Canada.

**Keywords:** Delphi, mixed-methods research, quantitative research, qualitative research, survey research

## Abstract

The use of the Delphi technique is prevalent across health sciences research, and it is used to identify priorities, reach consensus on issues of importance and establish clinical guidelines. Thus, as a form of expert opinion research, it can address fundamental questions present in healthcare. However, there is little guidance on how to conduct them, resulting in heterogenous Delphi studies and methodological confusion. Therefore, the purpose of this review is to introduce the use of the Delphi method, assess the application of the Delphi technique within health sciences research, discuss areas of methodological uncertainty and propose recommendations. Advantages of the use of Delphi include anonymity, controlled feedback, flexibility for the choice of statistical analysis, and the ability to gather participants from geographically diverse areas. Areas of methodological uncertainty worthy of further discussion broadly include experts and data management. For experts, the definition and number of participants remain issues of contention, while there are ongoing difficulties with expert selection and retention. For data management, there are issues with data collection, defining consensus and methods of data analysis, such as percent agreement, central tendency, measures of dispersion, and inferential statistics. Overall, the use of Delphi addresses important issues present in health sciences research, but methodological issues remain. It is likely that the aggregation of future Delphi studies will eventually pave the way for more comprehensive reporting guidelines and subsequent methodological clarity.

## 1. Introduction

Quantitative research encompasses a wide range of systematic and controlled designs, involves measurement and assumes that the phenomena can be measured.^[1,2]^ One of the most commonly used designs is experimental research, which aims to establish causality, involving careful selection of participants, random assignment, concealment, manipulation of intervention, and assessment of outcomes before and after treatment.^[3]^ When randomization is not possible, quasi-experimental designs can be used, which can be between-subject, which involves an intervention and comparison group (Sidani, 2014). It can also be within-subject, which involves 1 group of participants who receive the intervention, and undergoes repeated assessments.^[3]^ Next, correlational research is used when there is an inability to manipulate the independent variable, and aims to establish a relationship between 2 or more variables.^[4]^ Lastly, there is survey research, which is used to gain information about the relationship, incidence, and distribution of variables in a population.^[5]^

A survey is a series of questions or statements, called items, used in a questionnaire or an interview to measure the self-reports or responses of respondents (Privitera, 2018). On the other hand, survey research designs, which can be done in writing or orally, involve the use of a survey to quantify, describe, or characterize an individual or a group.^[6]^ Survey research designs can be divided into various forms, namely; Descriptive: gathers data related to attributes, behaviors and incidence of events; Longitudinal: a survey that is administered several times and; Correlational/comparative: surveys used to study and compare the relationships between variables.^[5]^ Surveys themselves should be valid and reliable to the research question, and a response rate of at least 40% is typically used, alongside precision, in which a margin of error of 5% or less is typically adopted.^[7]^ Overall, surveys are a commonly used and influential technique in health sciences research, especially if done through the Internet, which can make surveys cheap, anonymous, and have a worldwide reach.^[8]^ One commonly used research method, widely considered to be part of survey research,^[9–11]^ is the Delphi technique. Throughout this paper, the use of Delphi will be explored, key issues will be discussed, and recommendations will be proposed. This paper adheres to the criteria set by the SANRA, scale for the quality assessment of narrative review articles.^[12]^ A narrative literature search was conducted using keywords in the Pubmed, Medline and CINAHL databases, as well as Google Scholar, ranging from 2011 to 2021. Titles and abstracts were read to screen for relevance, and retained articles were read by full text. Of those that were read by full text, the reference lists were also assessed for any relevant articles. As this is a review of the literature, no ethical approval was obtained.

## 2. A brief overview of Delphi

### 2.1. History

Dealing with uncertainty is a perpetual component of the human experience, and throughout human history, researchers, theorists, and philosophers have attempted to perfect human mastery over it. In Ancient Greece, kings and generals have sought to face their uncertainties for their state and careers by consulting a prophet, the Pythia, or more commonly known as the Oracle of Delphi. The Oracle was known for her prophecies, which supposedly came directly from the Greek God Apollo. More than 2500 years later, during the height of the Cold War, the United States Army Air Corps also faced military uncertainties and consulted a think tank, the RAND Corporation. Using the name of the prophet from the ancient world, researchers from the RAND Corporation developed the Delphi technique, which involves recruiting several military experts, asking each expert about the probability, frequency and intensity of a potential Soviet attack, and then asking each expert to provide anonymous feedback, a process that is repeated until consensus is reached.^[13]^ Since then, this technique has been declassified and has evolved beyond its military applications into the different health sciences.

### 2.2. Basic tenets of Delphi

**Figure 1.**
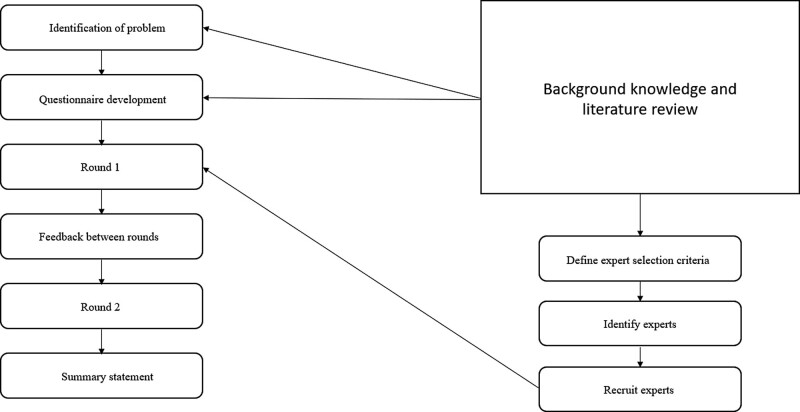
Sample conduct of a 2 round Delphi.

The Delphi technique is defined as the procedure of asking a panel of experts for their opinion on a relevant issue, summarizing and presenting their collective responses and repeating this process for a certain number of rounds (Fig. 1).^[14]^ Overall, there are 4 key features to any Delphi, namely anonymity, iteration, controlled feedback and the statistical aggregation of group response.^[15]^ Anonymity refers to the fact that participants should not know who else is involved in the study besides the researchers, and is achieved through the use of anonymized questionnaires^[15]^ and can be enhanced through assigning a unique confidential code.^[16]^ Also, anonymity from each other prevents undue influence by other participants potentially seen as superior or more expert than themselves.^[17]^

Iterations refer to the feedback process, which is viewed as a series of rounds allowing for participants to reassess their previous judgments.^[18]^ Controlled feedback refers to the fact that participants are informed of the responses of their anonymous colleagues, allowing for the vocalization of the collective opinions and judgments rather than the vocal few.^[15]^ Statistical aggregation involves the presentation of the statistical summary of the group response by the researcher, which are typically measures of central tendency,^[15]^ while also allowing for the final results to be amendable to statistical analysis, leading to a sense of quasi-objectivity.^[17]^

### 2.3. Consensus group techniques

The Delphi technique has been widely used as a forecasting tool to predict certain developments, build consensus around clinical issues and develop, describe and evaluate clinical guidelines or tools.^[19]^ Despite the diversity in its applications, consensus is a central theme and Delphi is considered a consensus group research method.^[20–22]^ Consensus group techniques involve obtaining the views of a group of experts, and aim to bring about consensus and agreement as outcomes.^[21]^ A key advantage of consensus group techniques is a balanced participation from participants through structured formatting.^[22]^

As a whole, researchers using consensus group methods assume that the views of a group are superior to that of an individual,^[15]^ consistent the psychology of the “wisdom of crowds.”^[23,24]^ To elaborate, “crowds,” or a collection of individuals, are often better at decision making than a single member of the group due to diversity of expertise, independent decision making, decentralized working conditions and aggregation.^[24]^ The Delphi technique incorporates these aspects, as there is an independence of decision making through anonymized questionnaires, decentralization through experts responding autonomously but sharing decisions through the researcher, aggregation through the researcher presentation of results and potential of having diverse expertise through appropriate recruitment criteria.^[25]^

Another consensus group method is the Nominal Group technique and the major difference between the Nominal Group and Delphi is that the Nominal Group is conducted in-person.^[20]^ Specifically, the Nominal Group technique involves participants receiving and reflecting upon a question, having the facilitator ask each participant to share their ideas to the group, generating a group discussion and lastly ranking the discussed ideas.^[26]^ In contrast to the Delphi technique, because the Nominal Group technique is done in-person, it is nearly impossible to be conducted anonymously and typically takes less than 2 hours to answer a single question.^[27]^ One of the primary advantages of using the Nominal Group is the ability to establish collaborative partnerships among the participants, and it is particularly well-suited for research designs where such partnerships are required, such as action research.^[21]^ Due to their respective differences, the Nominal Group technique is typically used in exploring consumer and stakeholder views, whereas the Delphi technique is used to create best practice guidelines and treatment protocols among healthcare professionals.^[22]^ Conversely, power differentials might have participants perceived as “weaker to rescind their actual views in face of the opinions of those perceived as “stronger.”^[22]^

The 2 most common forms of consensus techniques used are Delphi and Nominal Group,^[28]^ but there is also a third 1 that is occasionally used in health sciences research, the RAND Appropriateness method.^[29]^ In essence, the RAND Appropriateness method employs the Delphi technique through online or mailed questionnaires, and then uses the Nominal Group technique to discuss the findings generated during the Delphi phase.^[29]^

### 2.4. Different forms of Delphi

Currently, there is much debate surrounding the definitions of the Delphi technique. This adds to the methodological confusion, which is a major critique of the technique, related to the lack of methodological rigor, little existing guidance to help research and large variations in how Delphis are conducted.^[30–32]^ Below, there will be an outline on some of the most used methods, namely “classic Delphi,” “modified Delphi,” “policy Delphi” and “e-Delphi.” All these terms are often used interchangeably, and currently, no concrete definitions or guidelines exist to differentiate between these different, but related Delphi techniques.

The “classic Delphi” is a term which is mostly outdated, as it is now overwhelming being referred to as simply “Delphi”^[20,33]^ and is the most commonly used method. The classic Delphi is typically conducted for 2 to 3 rounds,^[34]^ and the first round involves a qualitative open-ended questionnaire or interviews to develop initial statements, generating a large amount of data (Trevelyan & Robinson, 2015). Subsequent rounds involve quantitative questionnaires, with central tendency and measures of dispersion to aggregate data.^[35]^

The “modified Delphi” is used in an incredibly diverse fashion, and almost no single modified Delphi is conducted in the same way. For example, in a widely cited study, Morisset, Johannson^[36]^ used a modified Delphi to identify diagnostic criteria for chronic hypersensitivity pneumonitis. In the first round, Morisset, Johannson^[36]^ used qualitative interviews and a literature review to identify initial items, and proceeded for 2 rounds using the classic Delphi approach. Subsequently, in the final round, researchers asked participants to use the newly conceived diagnostic criteria in a series of clinical vignettes, and to provide a level of diagnostic confidence. As for another influential example, Feo, Conroy^[37]^ used a modified Delphi approach to standardize the definition of “fundamental care.” For the first round, Feo, Conroy^[37]^ conducted an in-person interactive workshop during an academic conference on the topic of “fundamental care,” compiled the results of the workshop and went through 2 rounds of “classic Delphi.” Overall, most modified Delphis tend to involve an in-person aspect,^[14]^ such as interviews or focus groups,^[38]^ which are typically done in the first round. Nonetheless, the use of “modified Delphi” appears to be a blanket term for heterogeneous methods that are deviating from the “classic Delphi.”

An interesting deviation from the traditional Delphi approach is the “policy Delphi,” which is used in cases where consensus is not required, and dissensus is promoted.^[39]^ Thus, policy Delphis are not meant for decision making, but instead are useful as an analytic tool in policy issues.^[40]^ Although they are conducted in a heterogeneous fashion,^[41]^ policy Delphis tend to involve similar steps as the classic Delphi, in which issues are formulated, participants are asked of their opinion on the items, but also explore the reasons for disagreements and subsequently reevaluate the options.^[40]^

Lastly, eDelphi, defined as the classic Delphi but done completely online^[38]^ is increasingly being used as it offers unparalleled convenience, time and cost savings and allows for an unprecedented ease of data management.^[42]^ Specifically, the eDelphi will allow experts to participate regardless of geographic location or time zone and often leads to faster response times.^[17]^ Currently, the term “eDelphi is inconsistently used, as many researchers refer to their studies as simply “Delphi.”^[43]^ Therefore, due to the ubiquitous use of the Internet, most modern day Delphis are conducted as eDelphis, unless otherwise specified.

## 3. Importance to health sciences

The use of Delphi is important to health sciences research in several ways. As stated earlier, the Delphi technique is considered a manifestation of expert opinion developed via consensus. Expert opinion is considered to fall within the lowest level of evidence on the evidence pyramid, whereas the highest are systematic reviews and meta-analyses.^[44]^ Nonetheless, being “low” on the evidence pyramid does not mean that Delphi studies are without value or are considered low-quality research. Evidence-based medicine and nursing require a balance of studies involving the entire pyramid, and the dominance of 1 form would lead to discrepancies, confusion and an incomplete rendition of the phenomena under study. Furthermore, consensus methods are often used to determine the directionality of scientific research, unearthing what are the fundamental underpinnings of a field and are seen as foundational methodology upon which all other methodologies rest.^[25]^ Lastly, as the information stemming from experts tend to have direct and practical results, consensus by expert opinion allows for the easier generation of solutions for real-world problems. Despite its many advantages, there are several potential barriers to implementing a successful Delphi study. Being a form of survey research, conducting a Delphi can be a slow process, and it may take 2 to 6 months to complete a 2-round Delphi.^[45,46]^ A study involving closely contacting many participants over such a long duration may incur additional financial costs and resources, which the research team should be aware and adjust for. Thus, it is imperative for the researcher and/or Delphi coordinator the become familiar with the process, in order to have a streamlined process, which may avoid participant dropout and increase the overall study success.^[47]^

Delphi studies are useful to collect the first opinion on phenomena, and it is often used to examine an area with limited empirical research, and/or for where there are questions for which there may be no definitive answers.^[48]^ Relatedly, depending on the research team and research question, Delphis tend to quickly identify important points or issues, rapidly leading to conclusions.^[34]^ As one of the first empirical papers being published in an emerging field, it can subsequently influence a large body of literature. Therefore, the use of Delphi by various healthcare professionals can allow for opportunities to publish highly visible research. Furthermore, despite being used inconsistently,^[30–32]^ the Delphi is considered the most widely used consensus group technique.^[30]^ Thus, more Delphi studies should be conducted by nurses and other healthcare professionals to ensure future methodological clarity and conciseness.

## 4. Issue #1 – experts

### 4.1. What is an “expert?”

The Delphi involves the recruitment of a panel of “experts,” which the term “expert” is left open to interpretation. As a whole, an aim of the Delphi technique is to obtain high-quality responses from a select panel of experts, as opposed to getting a representative sample in traditional survey techniques.^[49,50]^ The Delphi technique uses a nonrandom sampling method, and aims to identify prominent, knowledgeable or representative people of the field under study.^[50]^ As Delphis use a nonrandom sampling technique, there is inherent bias in the recruitment process, since participants who are more interested in the topic are more likely to be involved for the various rounds.^[9]^ Unfortunately, participants cannot be selected randomly, due to the need to ensure “expertise,”^[50]^ a concept which will be elaborated in the section below.

Criteria used to define “expertise” is highly diverse, with examples being high educational attainment,^[17]^ part of the researcher personal network,^[51]^ years of clinical/practical experience,^[17]^ authorship in a peer-reviewed publication^[52]^ and membership in a professional association.^[53]^ Although educational attainment and years of experience are the most commonly used metrics to gauge expertise,^[35]^ there is an ongoing debate on the definition of expertise, and there are no current guidelines or standards on the selection of expert panel members. Commonly accepted requirements for participation in the expert panel include: experience and knowledge, willingness and capacity to participate, time to participate and adequate communication skills.^[35,54]^ Thus, researchers should strive to maintain a balance between these points. Additionally, a definition that is too stringent or specific would reduce the potential pool of participants, while a poorly defined definition can potentially affect the construct validity of the Delphi panel.

### 4.2. Number of experts

Another issue of ongoing debate is the number of participants to participate in a Delphi. The number of panelists can range from as few as 4^[55]^ to several thousand.^[56]^ Most commonly, Delphis tend to be within the range of 8^[57]^ to 20.^[25]^ Interestingly, a bootstrap study done by Akins, Tolson^[58]^ suggests that 23 participants lead to response stability within multiple rounds. Nonetheless, this is just 1 dated study, and more research is needed in studying the optimal panel size for Delphi studies. It has also been argued that findings will be more stable with larger sample sizes.^[25]^ To illustrate, a smaller panel of 10 experts can be highly unstable, as 1 person makes up 10% of the responses and thus is a major influence on the results of the study. With larger panel sizes, the individual expert influence on the study will be less, and findings will be more stable.^[25]^ On the other hand, researchers have found that large expert panels can introduce difficulties in data collection and management.^[18]^ Overall, Delphi panel sizes should be carried out with consideration to time and monetary constraints and ideally be between 8 to 23 participants.

### 4.3. Issues with expert selection and retention

Besides the number of participants, the choice of participants is also open to debate. Strict selection criteria and definitions of expertise lead to a more homogenous expert panel, whereas less restrictive definitions will lead to a more heterogeneous sample. Currently, it is recommended to have a heterogeneous sample in terms of expertise,^[25,31,55,59]^ as it leads to better performances and higher quality responses due to a wider range of perspectives.^[24,35]^ Furthermore, if the issue under study is used to inform broader policy or have a global relevance, then it may be optimal to have a more heterogeneous sample.^[60]^ However, it is also argued that heterogeneous panels can increase the complexity and difficulty of collecting data, reaching consensus, conducting analyses, and verifying results.^[61]^ This leads to a decision quality trade-off, as stability increases in tandem with sample size and heterogeneity (thus an increase in decision quality), but beyond a certain threshold panel size and heterogeneity, managing the Delphi process becomes cumbersome in return for marginal benefits.^[61]^ Although these issues are inherent with Delphi methodology and unique to the problem under study, a portion of these issues can potentially be mitigated by conducting pilot Delphi studies, or validating the results through triangulation with other techniques, such as qualitative focus groups.^[50]^

Next, attrition has been identified as a major issue being faced by Delphi studies,^[20,25,35]^ with attrition rates ranging from 0% to 92% for classical Delphis.^[62]^ As Delphis can have multiple rounds, more and more participants are likely to drop out through the subsequent rounds. This can be problematic, because increasing attrition over subsequent rounds can be due to participants with dissenting views to drop out, creating a false sense of consensus,^[20]^ leading to a form of response bias.^[50]^ Interestingly, Hejblum, Ioos^[63]^ found that there are lower response rates for Internet-based Delphis as opposed to a mail-in system, leading to Boulkedid, Abdoul^[31]^ to propose researchers to use a mixed Internet and mail-in approach. However, the applicability of the findings of Hejblum, Ioos^[63]^ can be critiqued due to its outdatedness, as the year of implementation is a strong predictor of mail-in-survey response rate.^[64]^

Overall, it is recommended to ensure that participants are fully informed of the study, including of the time commitments and researchers should maintain a short between-round time frame,^[17,31,35]^ while ensuring a presentable delivery of feedback. Also, sending in regular reminders to participants that each round is constructed out of their responses encourages interest, ownership and partnership.^[50]^ Due to the permeability and flexibility of the Internet, the use of Internet-based approaches for Delphi is recommended, and even though evidence (albeit outdated) suggests the contrary, it is without debate that Internet-based survey methods are much more cost-effective.^[65]^ Therefore, monetary funds could be redistributed to other aspects of the study, resulting in a decrease in attrition and an overall more robust study.

## 5. Issue #2 – data management

### 5.1. Data collection

Data is collected through Likert surveys, and summarized results from the previous round, alongside the participant own responses are presented to each participant. For Likert scales, typically 5 and 10 point scales are used^[66]^ and can be supported through graphical representation, such as bar graphs. Currently, there is debate around whether to include a midpoint (odd number of categories) or not (even number of categories).^[35]^ If there is a midpoint, there is a chance that participants may elect to choose the midpoint for: questions they have no opinion on, choosing a minimally acceptable response as soon as it is found and avoidance what appears to be the socially undesirable behavior of selecting a “negative” option.^[67]^ Nonetheless, the midpoint is useful for expressing neutral opinions, which is important for answering obscure and emerging topics,^[68]^ topics which are often studied by Delphis. Also, it has been argued that midpoints are not “dumping grounds” and instead the phenomenon can be attributed to a lack of question clarity by the research team.^[69]^ Therefore, researchers should carefully consider the clarity of their Likert questionnaire, which can be accomplished through pilot testing.^[38]^ It is also suggested to include as few items as possible within the survey, as a large amount of items is associated with lower response rates.^[70]^

### 5.2. Consensus defined?

As stated earlier, the aim of the Delphi technique is to achieve consensus. However, in a systematic review by Diamond, Grant,^[32]^ nearly every study uses their own standard for consensus, a finding that is also supported by a more dated review.^[31]^ Consensus can be defined in 2 ways, with the first being agreement with the statement and second being the extent participants agree with each other.^[71]^ Furthermore, there is stability, which measures if agreement is present throughout the Delphi process, or if it changed between rounds.^[71]^ With such confusing and ambiguous definitions going around, it is without a doubt that consensus is inadequately addressed by researchers, with roughly 26% of all Delphi studies not even defining consensus.^[32]^

Currently, the most common definitions of consensus are: percent agreement (i.e., x% with the same rating), measure of central tendency (i.e., median ≥7 on a 9-point Likert scale), proportion within a range (i.e., x% of participants scoring above a certain score on a Likert questionnaire) and dispersion of responses (i.e., interquartile range of 1 on a 5-point Likert scale).^[32,72]^ For the purposes of this paper, the term “definition” will be considered a measure, such as percent agreement or interquartile range. The term “level” will be considered the degree of the definition, such as 70 percent agreement, or an interquartile range of 2 on a 10-point scale. Despite this diversity of analytical measures and definitions of consensus, there are no currently agreed upon standards or guidelines for choosing 1 over the other.

### 5.3. Consensus – percent agreement, central tendency and measures of dispersion

Even with such diverse definitions of consensus, there are differing levels of each definition. For example, if going by percent agreement, then achieving a 100% agreement by all participants would be incredibly difficult. However, an unimpressively low percent agreement, perhaps around 30% to 50%, will be quite easy to achieve but renders the results of the Delphi less robust. Therefore, a balance is required, and the answer may lie within the importance of the research question, such as if it revolves around a life or death issue, then a very high consensus level will be desirable.^[73]^ If using percent agreement, then a level between 70% to 80% is usually adopted and widely considered to be rigorous.^[16,34,54,60,73]^ To summarize, researchers should aim for 70% to 80%, unless the research question is 1 that requires incredible precision, such as end-of-life guidelines, in which researchers should aim for 90% to 100%.

Other commonly used definitions are measures of central tendency, which include mean, median and mode and are recommended over percent agreement by Hsu and Sandford.^[18]^ However, Likert survey data are traditionally considered to be on the ordinal scale, and thus cannot be used for calculating the mean, as it can only be used for data on an interval scale.^[74]^ Therefore, it is recommended to avoid reporting the mean as a definition of consensus, and instead use median and mode. Furthermore, the median is less likely to be influenced by outliers, which can very likely occur if there is an expert with an extremely strong and divergent opinion on a certain issue. The mode is also used, but it can be problematic as it cannot capture multimodal data distributions, such as if an issue is perceived by some experts as moderately important, while by others as extremely important. Therefore, if using central tendency, it is recommended to solely use the median, while considering the mode as a form of secondary analysis.

One commonly used descriptive statistic and measure of dispersion is the standard deviation.^[66,74,75]^ The standard deviation is a statistic that measures the dispersion relative to the mean and is calculated as the square root of the variance. Thus, a smaller spread means a smaller standard deviation, which means that it is more likely to represent consensus. However, like the critiques of using the mean, the standard deviation is still sensitive (albeit less so) to outliers and cannot be used for ordinal data coming from Likert questionnaires. Another measure of dispersion is the interquartile range (IQR), which is defined as the amount of spread of the middle 50% of observations. The IQR is a frequently used metric for consensus, and is considered objective and rigorous by several authors.^[66,74,76,77]^ Typically, an IQR of 1 or less on a 4 to 5 item Likert scale and 2 or less on a 10 item scale can be considered as consensus, with the more points being on the scale, the larger the expected IQR.^[74]^ Therefore, researchers should elect to use the IQR to represent spread and consensus rather than the standard deviation.

### 5.4. Consensus to inferential statistics

Inferential statistics are statistics that help to establish relationships among variables and draw conclusion, which can be viewed as a measure of stability by various authors.^[35,74,75]^ The Chi squared test for independence is a nonparametric test by which 1 can assess whether there is a relationship between 2 variables. It has been proposed by Dajani, Sincoff^[78]^ as a method to check for the stability of the responses, but has been criticized by Holey, Feeley,^[75]^ who argue that Chi squared is instead testing for the independence of the Delphi rounds from responses obtained in them. Another inferential statistic is the Wilcoxon paired signed-ranks *t* test, which is the non-parametric alternative of the *t* test. Specifically, it compares the difference in responses to a survey item from 2 rounds, and assesses whether it is equal to zero.^[10]^ The Wilcoxon paired signed-ranks t-test can assess the degree of consensus, and thus is seen by several authors as a measure of stability.^[10,35,74]^ Overall, consensus is one of the most undefined aspects of Delphis, and stability is the most undefined aspect of consensus.^[10]^ Currently, it is unknown whether stability is a valid stopping criterion, or if inferential statistics truly represents stability, and therefore researchers should interpret the results of any inferential statistic in Delphi cautiously.

### 5.5. Consensus – lessons learned

Although consensus is a defining aspect of Delphi studies, over 70% of Delphi studies do not use the achievement of consensus as a stopping criterion, but instead only run for a predetermined number of rounds.^[32]^ If not using consensus as a stopping criterion, having a Delphi run for 3 rounds has been deemed to be optimal by several authors.^[35,61,79]^ One example of a 3 round Delphi is by Griffiths, who demonstrated high consensus (>90%) in identifying different treatment goals for pulmonary perioperative complications.^[80]^ Nonetheless, there can also be low percent agreement and consensus within a study, which would subsequently eliminate many of the proposed points, as seen in the 3 round Delphi done by Huijben in examining ICU care qualities for patients with traumatic brain injury.^[81]^ This can be advantageous for the research team, as it will allow for better logistical and time management, indirectly leading to cost savings. Additionally, forcing consensus by continuously conducting an indefinite number of rounds is counterproductive, because participants may eventually become frustrated and agree with each other to make it end.^[20]^ Generally, due to its simplicity and ease of planning, Delphi studies can be conducted with a predetermined number of rounds, ideally 3, without significant controversy. One possible exception to this will be for research questions which require a large amount of precision, such as the previously mentioned example of end-of-life guidelines. Also, setting a predetermined number of rounds is not an excuse to ignore or conduct a subpar data analysis, and researchers should always justify their choices.

In an interesting study, Grant, Booth,^[72]^ used available data sets from published Delphi studies and calculated final consensus based on several commonly used definitions with varying levels of stringency. In that study, the authors found that the percentage of items reaching consensus varied dramatically from 0% to 84% depending on the analytic procedure, leading to Grant, Booth^[72]^ to caution readers against potential data mining and selective reporting of consensus. Relatedly, there is always a chance that consensus represents collective ignorance as opposed to wisdom.^[60]^ Also, even though anonymity prevents any explicit form of domination,^[17]^ inherently weaker-willed members may be inclined to change their opinions due to the sole desire to conform, while inherently strong-willed participants may continue to rigidly hold onto their views.^[73]^ Therefore, even if consensus is “achieved,” it should be carefully interpreted, and it does not necessarily mean that the statement is “correct.”^[73]^ As a whole, it is suggested that researchers should always report a priori the consensus definition and level, and ideally explain their reasoning for choosing the various definitions and levels. Readers should always critically appraise the methodology, design and results, and be aware of the inherent weaknesses surrounding the Delphi technique.

## 6. Conclusion

To conclude, the use of the Delphi technique is useful in answering several critical problems within the different health professions. The combination of anonymity, iteration, controlled feedback and the statistical aggregation of group response makes it ideal for addressing emerging and unknown topics and forecasting issues of importance in medicine, nursing and others. Furthermore, as a form of expert opinion consensus technique, it has the potential of being one of the first peer-reviewed publications in a new field, subsequently influencing a large body of literature. Lastly, Delphis are often cost-effective and able to gather experts from geographically diverse areas. However, there continues to be methodological uncertainty and a lack of clear guidelines. More precisely, there are ongoing debates about the definition of expertise, how many panel members to recruit, definitions of consensus and issues with using different forms of statistical analysis. Accordingly, the preliminary reporting guidelines set out by Diamond, Grant^[32]^ can be used as a foundation for future Delphi research. Naturally, it is likely that the aggregation of future Delphi studies will eventually pave the way for more comprehensive reporting guidelines and subsequent methodological clarity.

## Acknowledgements

This paper is dedicated to the memory of San Seungwoo Hong.

## Author contributions

**Conceptualization:** Zhida Shang.

**Formal analysis:** Zhida Shang.

**Investigation:** Zhida Shang.

**Methodology:** Zhida Shang.

**Validation:** Zhida Shang.

**Visualization:** Zhida Shang.

**Writing – original draft:** Zhida Shang.

**Writing – review & editing:** Zhida Shang.
